# Normalizing toxicity: the role of recommender algorithms for young people’s mental health and social wellbeing

**DOI:** 10.3389/fpsyg.2025.1523649

**Published:** 2025-11-06

**Authors:** Kaitlyn Regehr, Caitlin Shaughnessy, Minzhu Zhao, Idil Cambazoglu, Alfie Turner, Nicola Shaughnessy

**Affiliations:** 1 University College London, London, United Kingdom; 2 University College London, University of Kent, Canterbury, Kent, United Kingdom

**Keywords:** misogyny, social media, algorithm analysis, mental health, neurodiversity, digital literacy

## Abstract

This article explores how social media recommendation systems shape the digital consumption practices of young people and the potential implications for mental health and wellbeing. It examines how the consumption of increasingly radical content, with a focus on gender-based violence and misogyny, is presented on young people’s feeds in increasingly high dosages, which has significant implications for young people’s social development. Employing a mixed-methods approach, this research draws on three data sources: (i) long-form interviews with young people, (ii) algorithmic analysis of over 1,000 social media videos, and (iii) roundtable discussions and interviews with school leaders from across England and Wales. These methods were used to triangulate how digital environments encourage and normalize harmful ideologies, normalizing radical content, and the affective impacts of this content on young people’s wellbeing. The study presents three main findings. First, recommendation systems amplify and subsequently normalize harmful ideologies, increasing users’ exposure to radical material. Second, misogynistic content is often presented as entertainment, which enables it to gain high levels of traction on social media platforms. As a result, hateful ideologies and misogynistic tropes appear in young people’s behaviors, which may have significant impacts on their mental health and peer relationships. Our findings suggest the need for a significant change in approaches to digital literacy, education and policy to support young people’s wellbeing and social development in digital spaces.

## Introduction

Economist Ben Marder has classified contemporary young people as ‘social natives’, having predominantly experienced their formative years engaged with the social, participatory web and have interacted with smartphones and tablets from birth (Ben Marder, 2020). Unlike their predecessors (‘*digital natives’*: 25–34-year-olds, Prensky, 2001), when it comes to news, access and attitudes, social natives increasingly use social media (Instagram, TikTok and YouTube) not just as a way to socially interact, but as a way to consume news and access information. Whilst forming communities through social media can have a positive benefit for young people, affirming social identity and creating connection, particularly for displaced, marginalized, or disenfranchised young people (Ko and Kuo, 2009; Davis, 2012), some aspects of online communities can also be problematic (Allen et al., 2014). There are concerns regarding how the affordances of social media platforms are increasingly exposing young people to untrusted, unverified, and radical material, which is subsequently impacting their peer relationships and wellbeing (e.g., Amnesty International, 2024; Reset Australia, 2022).

While the risks that these types of platforms present in terms of disinformation and radicalization has been highlighted by many across both media and academia (Floridi, 2016; Lazer et al., 2018), longitudinal, psycho-behavioral research on the impacts of digital engagement on adolescent development, focusing specifically on the long-term mental health impacts to young people, is still emerging (Odgers and Jensen, 2020). As Candace Odgers (2024), along with Jensen et al. (2019), has emphasized, digital technologies may be amplifying many of the existing offline risks regarding anxiety, self-harm, and radicalization that young people experience in their teenage years. As Przybylski et al. (2020) found, the associated links between digital technology use and wellbeing also have a ‘u-shaped’ relationship, with poorer wellbeing outcomes for those at either extreme—either very high or very low digital screen engagement. For researchers, there is a need to understand how particular vulnerabilities of young people intersect with their online experiences. Within wider society, however, there are growing assumptions that the rise in mental health concerns among young people (Banks et al., 2023) is strongly linked to smartphone use (e.g., Haidt, 2024, “The terrible costs of a phone-based childhood”), but it is likely the picture is more nuanced. For example, we know that online behaviors and risks also often mirror offline vulnerabilities (Odgers and Jensen, 2019).

The growing prominence of radical material has heightened existing worries about the impact of digital usage on adolescent development and their peer-to-peer interactions (Lewis-Kraus, 2022). Young people are vulnerable to online echo chambers, and the circulation of electoral fake news, mis/disinformation, and polarization toward political extremes (Floridi, 2016; Krasodomski-Jones, 2016; Lazer et al., 2018). In particular, concerns have been raised regarding how digital access is impacting peer relationships (Children’s Commissioner, 2023). For example, the online initiative ‘Everyone’s Invited’ sparked a national dialogue concerning the perpetuation of harassment and sexual abuse among young people in schools in the UK, with many pointing to the role of digital sexual violence in enabling and amplifying this phenomenon (Children’s Commissioner, 2023). According to research conducted by the Children’s Commission, a significant majority, 79%, of young people had been exposed to violent pornography before turning 18, and those who frequently consumed pornography were more likely to participate in sexual violence within their own relationships (Children’s Commissioner, 2023). A rapid report released by Ofsted in 2021 on sexual abuse in educational settings underscored the prevalence of the problem, with nine out of 10 girls reporting they had experienced some form of sexist abuse or image-based sexual harassment (IBSH; Ofsted, 2021). Concurrently, research has also revealed widespread digital sexual violence, with the majority of young women and girls reporting instances of IBSH and Image-Based Sexual Abuse (IBSA) (Ringrose et al., 2021). In response to these findings, there were calls to investigate the evolution of gendered dynamics such as ‘lad banter’ in online environments. As part of these recommendations, Ringrose et al. (2021) emphasized the role of digital literacy and the importance of fostering trust and openness between young people, parents, and teachers regarding online activities. As tech companies grow in power and profit and young people spend an increasing number of hours online, there is a critical need to interrogate the exact processes and affordances of these platforms, examine the ways in which types of negative content are delivered and consumed, and their impacts upon young people’s behaviors and wellbeing (Allen et al., 2014). This article takes the case study of online misogyny to consider wider questions regarding the affordances of social media and young people’s wellbeing: How do algorithmic processes popularize and normalize negative, toxic material, and how might this impact young people’s wellbeing?

### Misogynistic extremism in mainstream youth cultures

Concerns around digital misogynistic extremism has over the past 5 years, focused on a small, subculture of people (typically young and male) identifying as Incels or ‘involuntary celibates’, who feel left out of romantic relationships and often turn to the digital space to express their frustration, anger, and hate against women (Hoffman et al., 2020). ‘Incel’ denotes a demographic of young individuals who perceive themselves as excluded from romantic relationships as well as from broader societal inclusion. Typically, these individuals turn to online platforms to express sentiments of frustration, anger, and occasionally, a longing for retaliation (Regehr, 2022), with some isolated cases having been linked to incidents of mass violence, particularly in the United States and Canada (Horne, 2024). Much of this discontent is directed toward women, and as Diaz and Valji (2019) have argued, there is a strong link between Incel misogyny and acts of physically violent extremism (Díaz and Valji, 2019). Researchers exploring Incel culture have emphasized how these individuals often feel like outsiders and struggle with social anxiety (Daly and Reed, 2022; Regehr, 2022; Speckhard and Ellenberg, 2022). Many have significant mental health concerns, including depression and suicidal ideation, as well as bipolar, borderline personality disorders or autism (Johanssen, 2023, p. 195), and often describe previous psychological trauma of bullying or persecution.

Within these communities, Incels have historically embraced elements of ‘nerdom,’ or what Massanari (2017) categorises as ‘geek masculinity’, whilst also adopting the rhetoric of the oppressed or marginalized (Ging, 2019). Incel communication is often maintained through a use of community-specific ‘sensational language’ (Daly and Nichols, 2023) mixed with memes, satire, irony, and burlesque humor (Cottee, 2020). As Regehr (2022) emphasized, technology plays a key role in facilitating processes of indoctrination that transformed into misogynistic extremism (Regehr, 2022). Over the past 5 years, the affordances of online spaces have accelerated this, enabling content that was once relegated to marginalized corners of the internet to be perpetuated more widely. This has had the effect of creating larger, more empowered and connected ‘Incel-aligned’ communities. Incel, or what it used to be, was fundamentally about being alone—characterized by feelings of loneliness and isolation (Ging et al., 2020). As described by one participant, ‘they are missing that sense of community. There’s talk about rejection... Although it appears to be less about romantic rejection and more about social rejection’ (Regehr, 2022, p. 144). The ability to now connect to numerous young men online represents a notable development. While Incel 1.0 revolved around being an outsider, we prepose a new typology of Incel—which we term ‘Incel 2.0’, by way of contrast, emphasizes camaraderie, and perpetuates across broad swathes of popular social media. Employing typical Incel and misogynistic rhetoric and tools, Incel collectively articulates contemporary frustrations of young men, and in this way, ideas of Incel are now saturating mainstream youth cultures. On popular social media, it is evident that this type of Incel 2.0 material is being normalized and presented as entertainment. As we will discuss in this article in relation to our interviews with school leaders and safeguarding leaders, the migration of Incel rhetoric on to mainstream platforms like TikTok has led to a popularization of misogynistic culture across a much wider subsection of youth.

We prepose that Incel 2.0 encapsulates the shifting landscape within Incel culture, as it increasingly permeates broader and more mainstream youth cultures. The affordances of popular social media have resulted in the transfer of toxic material from alternative forums like 4Chan, 8chan, or Discord onto more popular platforms, such as TikTok and YouTube (Nagle, 2017). Influencers, or ‘In (cel)fluencers’ (such as Andrew Tate and Jordan Peterson), have promoted misogynistic messaging specifically at young boys through the normalizing of male victimhood narratives, using techniques of banter and shock in order to gain widespread appeal (Haslop et al., 2024). These narratives draw on similar themes observed in manosphere content, with an emphasis on ‘facts’ and ‘science’ to ‘justify misogynistic and gender essentialist philosophies’ (Ven and Gemert, 2022), alongside pseudo-incel content that relies on extreme rhetoric and the victimhood of men, both of which appear to appeal to social media algorithms (Deem, 2019; Haslop et al., 2024). This has resulted in the normalization of significant volumes of hateful material, including extreme and violent viewpoints, among young people through their use of social media platforms. For instance, a 2022 report released by Reset Australia illustrated how algorithms propagate misogynistic and anti-feminist content, including that of Jordan Peterson, to users’ recommended video lists in a short span of time (Reset Australia, 2022, p. 6). Furthermore, Amnesty International (2024)found similar patterns regarding the easy access and volume of self-harm material available to young people on TikTok and Instagram.

The current study sought to trace how toxic content online was becoming embedded within mainstream youth cultures. It began with interviews of young people involved in both consuming and creating radical online misogyny within radical Discord servers, and across the course of the research, we traced how the same discourses and memes were becoming embedded within material on easily accessible platforms (such as TikTok) and algorithmically offered to young people. To explore the affective impact of this material within schools, we subsequently interviewed school leaders about their experiences regarding how this material was manifesting in behaviors in school settings. Taken together, these tripartite streams of information led us to uncover key factors regarding how the affordances of social media algorithms were popularizing and normalizing negative, toxic material, and the subsequent impacts of this on young people’s wellbeing. This article discusses initial results in relation to this fieldwork, policy responses and considers future directions for how to approach the questions regarding social media and its impact on young people’s wellbeing.

## Materials and methods

The research utilized a mixed-methods approach, synthesizing data from three data sources: (i) in-depth interviews with 10 young people engaging in online misogyny, (ii) analysis of over 1,000 social media videos, and (iii) roundtable discussions with school leaders to investigate the perpetuation of gender-based violence and misogyny among young people. This three-stage mixed-methods approach enabled not only the rigorous analysis of online content presented to young people but also the tracking of the cognitive and affective changes of that content from the perspectives of young people themselves and those who work with them. Ethical approval was sought from the [redacted for peer review] Research Ethics Committee prior to fieldwork, and all participants gave their informed consent to take part.

(i)  Interviews

We undertook 10 long-form interviews with young people who had seen, had been algorithmically fed, or who were engaging with online misogynistic content. These informants do not necessarily identify themselves as “misogynistic,” but rather had various levels of engagement with topics related to lamenting women’s and minorities’ upward mobility in society, relationships, and generalized misogynistic material. Many of these forums served as gateways, where, upon joining, the researcher received links to private servers where users openly shared and discussed violent, misogynistic content.^1^ Working with a male researcher and documentary maker who had previous experience of, and contacts within, these online groups, participants were recruited through Discord forums. Discord was chosen as the most appropriate platform for recruitment as it is now the chosen forum by these groups for invite-only servers and discussions of misogynistic content.^2^

Upon gaining acceptance into these channels, the researcher would initiate conversations with participants over a period of several days. Initial contact typically involved a voice chat to introduce the research and to gain insight into their lives. These discussions often centered around engagement online, with questions including the types of forums to engage with, and the length and types of engagement. Questions also centered around experiences with online misogyny, e.g., How they were introduced to the content and how it made them feel.

Participants were primarily recruited through a snowball sampling approach on private forums. Once a participant responded and agreed to an interview, they often invited the researcher into a smaller, more intimate server used by a close-knit group, allowing for further engagement. Interviews were conducted anonymously over the phone and typically lasted 90 min.

(ii)  Online analysis

In order to gain deeper insights into the widespread dissemination of online misogyny across online platforms, we conducted online fieldwork aimed at examining the paths through which individual users encounter this online material.

The ‘For You’ page on TikTok is tailored for each individual account, presenting videos and content from accounts followed by the user, as well as broader recommendations based on the user’s inferred interests. These interests are determined by extensive data collected by the algorithm, including factors like the duration of time spent on particular videos, user interactions such as likes or comments, and the utilization of relevant hashtags in user-generated content. Given the highly individualized experience of TikTok, our study introduced ‘Archetype Modeling Methodology’ to investigate how algorithms influence exposure to misogyny content for TikTok users. This approach is modeled on similar methodologies employed by organizations like Amnesty International (2024) and Reset Australia (2022) to study how recommender systems introduce content to users online. These approaches let the algorithm ‘run’ unaided on blank accounts, enabling researchers to track what was recommended to accounts based on viewing and search history.

In our study, four archetypes were developed to examine how initial interests in key thematic areas influence the presentation of misogynistic content to young people through the TikTok algorithm. These archetypes were constructed based on the thematic analyses of the interviews with individuals engaging in misogyny, incorporating specific terms and interests derived from typical users. After two authors (CS, IC) undertook a thematic analysis of the interviews, a reflexive, collaborative thematic analysis workshop was conducted with all authors, where the thematic analysis was presented and subsequently discussed to identify vulnerabilities and decide on criteria for archetypes. This was then developed and confirmed with all members of the research team prior to implementation.

Each archetype embodies particular characteristics and vulnerabilities observed within these online communities (see Table 1). Each archetype was run on a blank account across 7 days to explore how and to what extent videos presented through the algorithm aligned with the respective archetype’s preferences and interests.

**Table 1:** Description of archetype criteria.

Archetype	Description
Archetype 1	Individuals who are experiencing a sense of loneliness. They might have low self-esteem or low self-efficacy, have experienced bullying, and have high rates of internet use. May be ‘NEET’ (not in employment, education, or training). **Loneliness:** This might include content that addresses feelings of loneliness, talks about bullying or school absence. This might include personal stories of resilience from bullying. **Anti-establishment:** This might include content that criticize the capitalist system, capitalists, and politicians. This might include videos discussing socio-economic issues, inequality, and unemployment. **Male victimhood:** Victimhood narratives and discussions regarding perceived societal biases against men. Content emphasizing the dominance of men, with rationalization, that is based on pseudoscience and statistics. Essentialistic explanations of gender hierarchy. **Masculinity and power:** Inspirational stories of men, Career and Job Search Tips on how to make money.
Archetype 2	Individuals who are more focused on the development of mental health knowledge and neurodiversity. They might seek out content that has an overtly negative focus on negative neurodiverse experiences. **Neurodiversity**: Content including a focus on personal stories and empathy about mental illness/health, psychology, autism, ADHD, or dyslexia. **Self-improvement narratives:** Inspirational stories of people to achieve success, believing in yourself, content that has clear-cut/black and white thinking and journeys out of adversity. **Negative societal outlook**: Content providing negative opinions about society, videos criticizing the issues of society, may include extreme perspectives and conspiracy theories.
Archetype 3	An individual may be interested in male enhancement, fitness, and bodybuilding, as well as general dating and relationship advice. **Male appearance and self-improvement**: ‘Looksmaxing’, appearance enhancement, fitness, and bodybuilding, also videos presenting strong men, or boxing. Content related to self-improvement, confidence, and sexualisation. **Dating advice**: Videos related to dating tips, dating apps, and related content. This may include videos related to porn or sex. **Men’s rights**: Masculinity and power, presenting manhood, videos related to violent or explicit content.
Archetype 4	An individual who is more aware of some generalized men’s rights content, and may already be fatalistic, angry, and cynical. High levels of internet use. **BlackPill**: Content that emphasizes looks-based attraction, related to appearance enhancement, fitness, and bodybuilding. **Mental Health**: content related to self-harm and depression. Videos involving homophobia and transphobia. This may include videos that promote fatalism and determinism. **Negative societal outlook**: Content providing negative opinions about society, videos criticizing the issues of society, may include extreme perspectives and conspiracy theories. This may include right-wing extreme views and extreme violence. Videos discussing activism, protests, and political and social activism.

**Figure 1:**
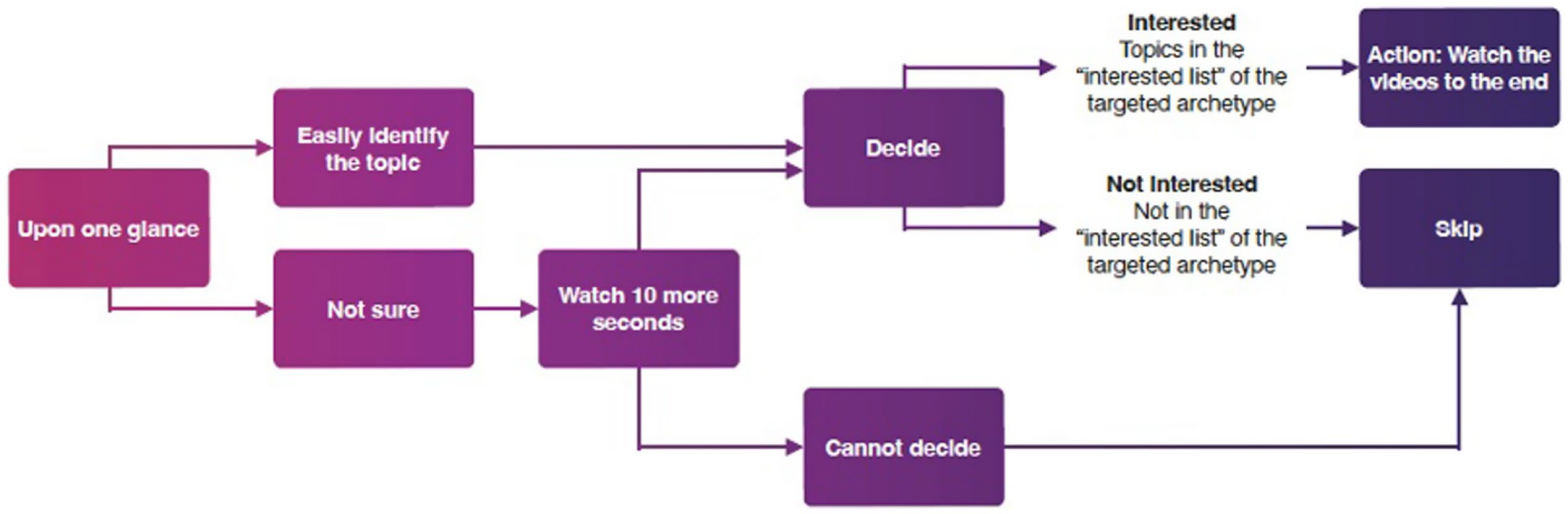
Diagram of TikTok procedure flow.

After identifying the interests of the four archetypes and outlining the procedure for video viewing (see Figure 1), each archetype was sequentially run for 7 days, spanning from August 22nd to September 27th, 2023, on four factory-reset iPads. No identifying information was provided to TikTok during the account creation process. Due to ethical considerations, especially concerning interactions with users under the age of 18, the only activity conducted on the archetype accounts was video watching. No proactive actions such as liking, commenting, or searching were performed. For each archetype, a researcher spent 1 h per day watching videos on TikTok continuously for 7 days. Seven days were set as the window for watching and collecting videos, given the limitations of analyzing vast quantities of video data, and the observation that after 5 days of watching, the algorithm had reached saturation in the typologies of videos presented.

If the videos recommended by the algorithm were deemed to align with the preferences of the respective archetype, they were watched in full, and the shared link was recorded. All relevant videos from each day were documented, and videos from day 2, day 5, and day 7 were subsequently thematically coded. In order to observe differences between three points in the week. These were then mapped through data visualizations to explore how different themes co-occurred.

Videos were initially collected and coded by one researcher (MZ). All links were kept, and random spot checks were done by other members of the research team to confirm alignment with the archetype criteria. Viewing decisions were only based on viewing of content—no hashtag or other criteria were used, although these data were documented in the larger dataset. If there was any concern or lack of clarity about a potential video, this was either skipped in the initial watching stage or removed from the dataset.

(iii)  Expert consultation

To understand further how access to this material was manifesting in the relationships and behaviors of young people more widely, we undertook nine expert interviews with senior leadership and safeguarding teams from schools nationwide. Subsequently, in partnership with the Association of School and College Leaders (ASCL), we ran two wider events: a roundtable focus group in October 2023, which was attended by 25 senior leaders, and a workshop session at the ASCL Safeguarding and Pastoral Conference, attended by over 100 safeguarding leaders from across the country.

### Analysis

**Figure 2:**
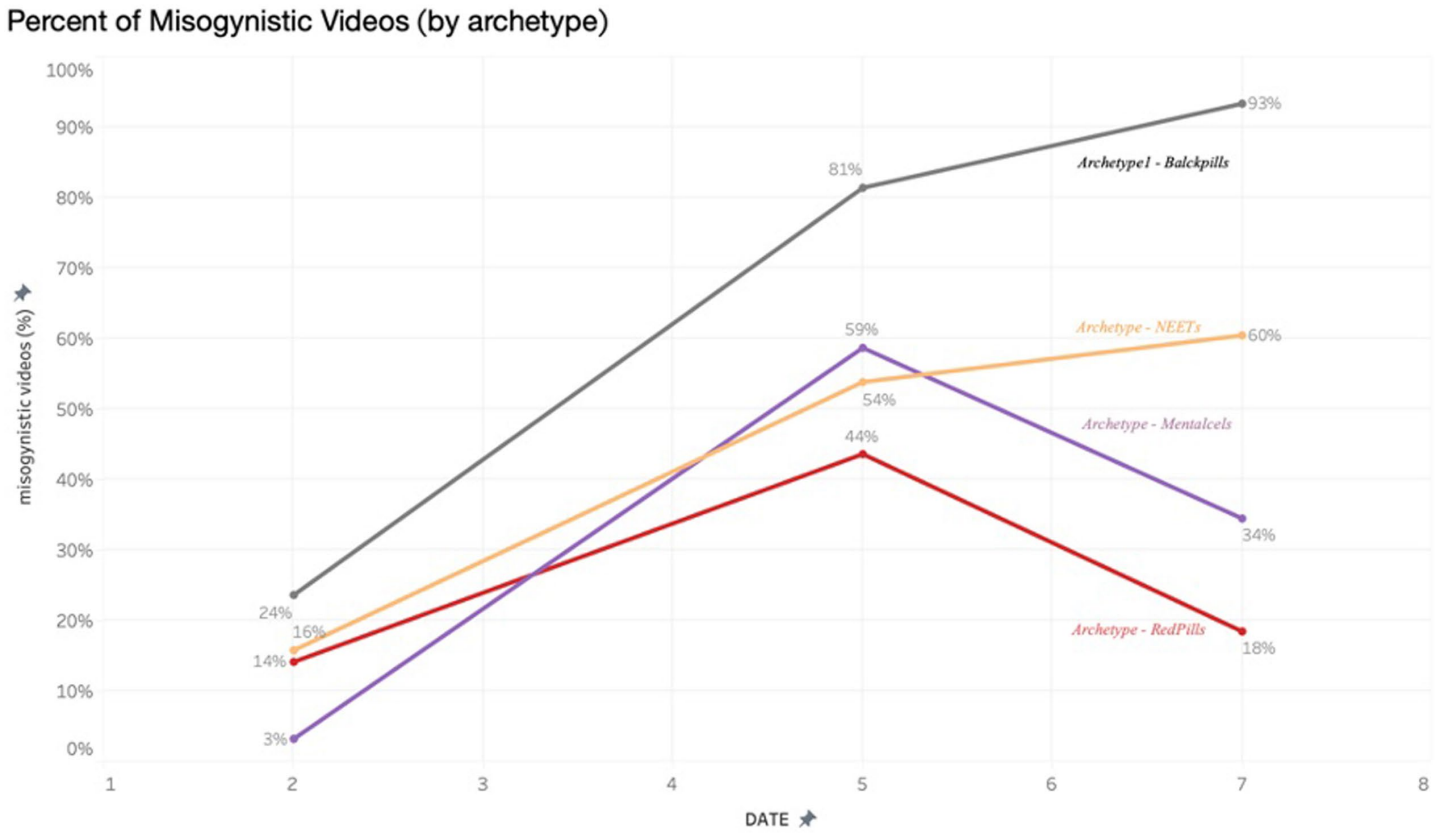
Percent of misogynistic videos by archetype.

**Figure 3:**
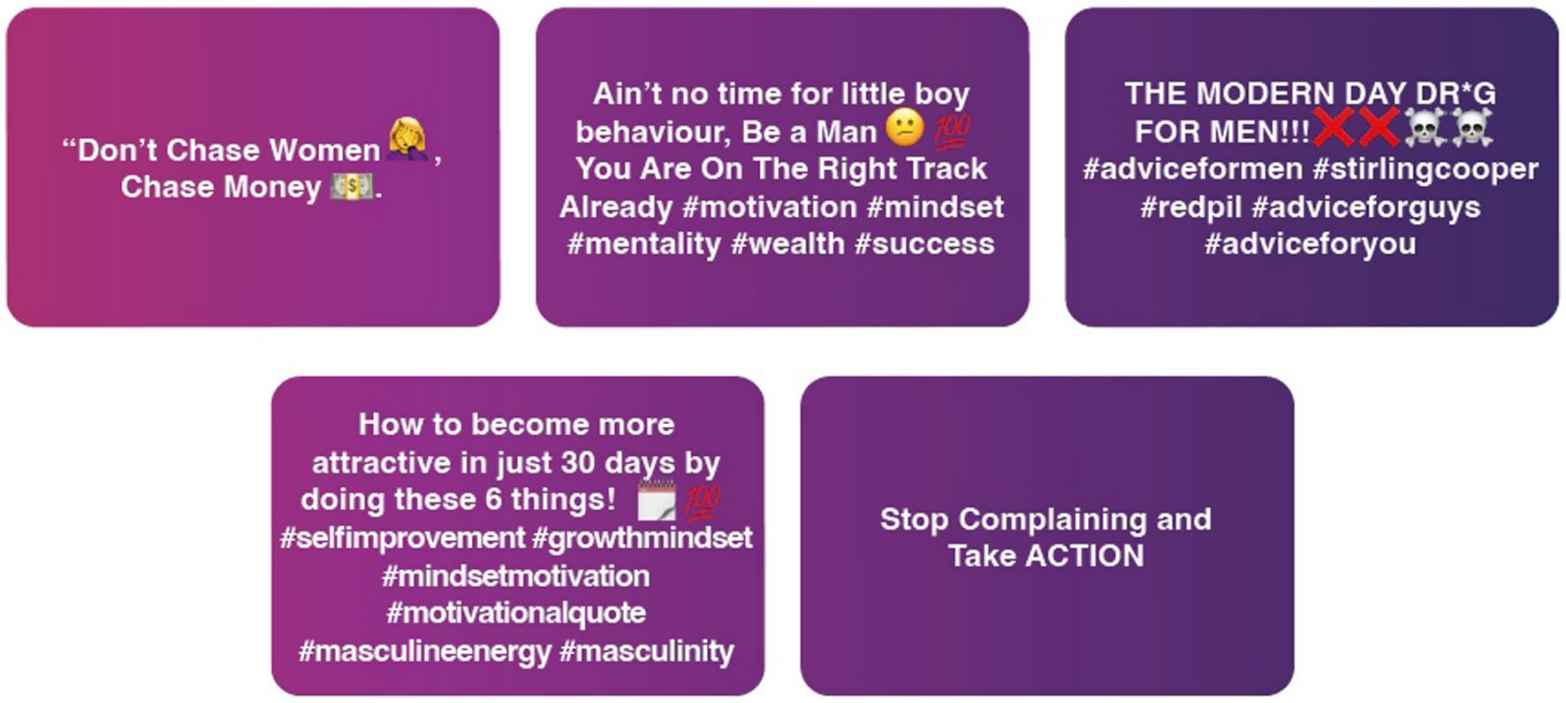
Exemplar posts of self-improvement and masculinity content.

**Figure 4:**
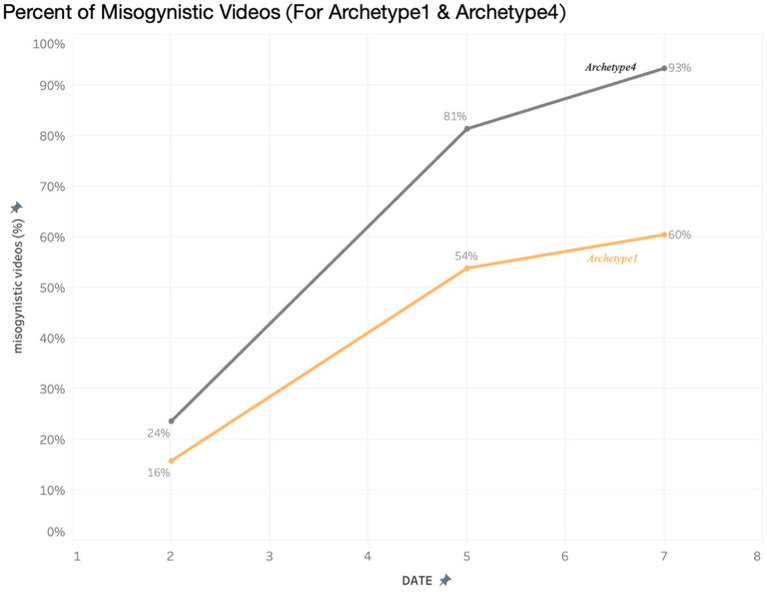
Percent of misogynistic videos for archetype 1 and 4.

Qualitative data from individual interviews, expert interviews, and roundtable focus groups were thematically analyzed alongside the collected videos according to thematic analysis. Following the five-step process, as outlined by Braun and Clarke (2006, 2022), the research team undertook a flexible and iterative process to uncover insights from the collected qualitative data. This process included first identifying initial codes for interview and video data individually, then drawing those codes into wider patterns and themes. Initial coding was done by CS, IC (qualitative interview and focus group data), and MZ (video data) independently. CS second coded both IC and MZ’s analysis, and any disagreements were discussed and referred to KR and NS if needed. Early in the process, the coding authors came together to discuss the initial articulation of themes. These themes were then compared alongside data visualizations from the coded videos obtained during the online fieldwork (see Figures 2–4), expanding our analysis and understanding of how different themes co-occurred and intersected. Subsequent reflexive analysis workshops with KR, AT, and NS offered further insights into the themes and identified higher-level themes to provide an overview across the three datasets.

Some themes drew more clearly from some categories of data than others. For example, themes that explored the online environment and content were more prominent in the video and interview data with young people (Themes 1 and 2). Themes and codes that referred to the impact of online content were more heavily represented in the accounts from young people and teachers (Themes 2 and 3).

In this iterative approach, the visualizations from the online fieldwork further shaped the analysis, helping to identify how key themes became more common and embedded within the archetype experiences as they spent more time online. As emphasized by Charalampidi and Hammond (2016), the utility of mixed-methods approaches to analyzing online activity enables a more comprehensive picture of the experiences of online participation. As with this study, our methods of analysis do not constitute an ‘off the peg’ approach (Charalampidi and Hammond, 2016) and are uniquely tailored to understand and interrogate the material, experiences and algorithmic processes through which targeted content is delivered to individual accounts through experiences that are highly individualized and often obfuscated by the algorithms of the social media companies themselves.

## Results

### Theme 1: the normalizing of radical ideology

#### Increasing radicalism

Through the algorithm analysis, it was observed in real-time how the algorithm directly amplified radical content. The methodological combination of interviews with young people and teachers, as well as the analysis of online content, enables the tracking of the cognitive and affective changes in the minds of the recipients. Following a 5-day period, all archetypes experienced a significant growth in misogynistic content on their respective ‘For You’ pages, with instances of ‘misogynistic content’ escalating from 13 to 56%. Particularly pronounced increases were noted among archetypes emphasizing loneliness (Archetype 1) and radicalism (Archetype 4).

The increasingly radicalism threading of this content was subtle, as users ‘microdose’ on progressively more negative, toxic material. Initially, the content presented to the archetypes often acknowledged and empathized with themes regarding social difference within men, delving into discussions regarding loneliness or personal growth. However, with prolonged usage, this content increasingly shifted toward expressions of anger and assigning blame. Of the four archetypes, there was a distinct divergence in the type of content presented after day 5. Archetypes 1 and 4 saw a significant continued rise of misogynistic content over the 7 days.

For Archetypes 2 and 3, content that began as overtly misogynistic began to be replaced by softer, sanitized forms of ‘toxic masculinity’, such as ‘how women think’ and female narcissism.

Alongside other mental health-related content, the material encountered by the archetypes evolved from addressing issues of loneliness and social disparity to expressing anger with a pronounced misogynistic^3^ slant (Regehr, 2022). This parallels much of the existing research on Incel communities, where the utilization of ‘therapeutic’ rhetoric (Johanssen, 2023) serves to reinforce group cohesion, enabling members to collectively articulate their shared experiences of mental health struggles and social isolation, including sensations of being ‘left out’ (Kay, 2021) in online environments. As one young person reported, TikTok was a fertile ground for this material to be hosted ‘TikTok has the most videos of any place…because it’s easy to consume content and easy to create content that will not get banned. Go on TikTok and search Black pill, Red Pill anything like that and you’ll get tonnes of results’ [Young person, P6]. The collective reinforcement of increasingly radical ideology was widespread, where online echo chambers reinforce a sense of shared hopelessness regarding life possibilities, contributing to their vulnerability to extremism and hate ideology. This creates a form of negative cognitive scaffolding in which the community echo chamber creates a form of emotional contagion, reinforcing and validating identity hostilities as experience so that extremes of thinking and feeling become increasingly entrenched as belief. As another participant reflected, ‘It’s very fun and appealing to have belief in things (...) you are just a part of something’ [Young person, _3]. This echoes, as Tirkkonen and Vesterman (2023) noted, ‘the social interaction patterns in Incel communities provide a feeling of belonging at the expense of regaining hope’.

#### Patterns of indoctrination

In our discussions with young people about their own process of radicalization, they were reflective about their own journeys down the ‘rabbit-hole’, directly identifying and demonstrating an awareness of how they are sucked into these belief patterns but are nevertheless, unable to break the cycle, committed to increasing consumption of content in which they had found their own sense of community and belonging, as one participant noted:

It becomes dangerous when you’re on something [for] a specific niche. Because you’re getting everyone who’s in that same mind set digging each other up more and more and more and more until a point where like… you no longer your original yourself by the time you got to the end of it … it just depends how easily, emotionally manipulated you are in that sort of situation’ [Young Person, P1]

Another described their own exposure and subsequent ‘rabbit-hole’ via algorithms, which resonated and explained their own sense of isolation:

I was on YouTube one day and I got recommended a video … I clicked on it, it was expressing black pilled ideas and stuff … it was a rabbit hole and I related to the message a lot growing up … it was just weird how that was recommended to me but I mean the video I think it has a million views right now. So obviously was it branched out to a lot of men …but that message did reach a lot like me, I've never been exposed to such idea before. It felt just like a stone being lifted off your back … just like being saved [Young person, P8].

To further investigate how the algorithm reshaped mental health narratives of loneliness or isolation into misogynistic or toxic masculinity-themed content, we examined the evolution of different themes throughout the duration of our fieldwork. For Archetype 4, we observed a transition in video content from focusing on loneliness, the challenges of life, and pressures faced by men in the initial days, to topics such as relationship advice, masculinity, and understanding women. However, these themes were all interpreted through a distinctly misogynistic perspective.

**Figure 5:**
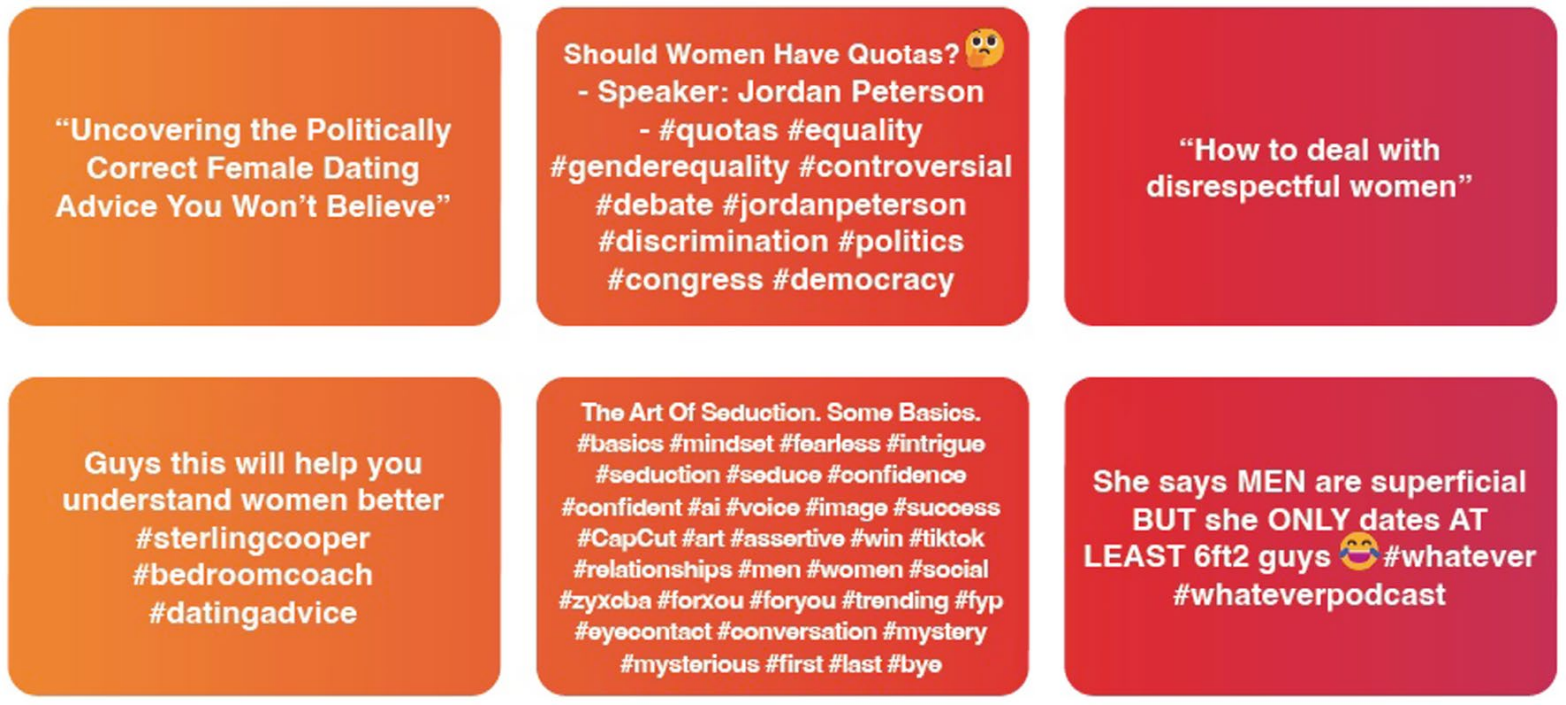
Posts discussing dating and relationship advice.

This indicates that individuals seeking content to cope with mental health issues or feelings of loneliness are susceptible to being exposed to narratives that place undue blame on women for their circumstances. As is evident in the visualizations of Figure 5, a hypothetical feed that was once focused specifically on issues of loneliness or self-improvement has moved to focus more specifically on ‘pressure on men’ and ‘masculinity’, but which also co-occurred with content regarding relationship advice and how women think. By day 7, the content of the videos now predominantly focuses on misogynistic content, particularly relationship advice. As one young person reflected, ‘It’s really getting targeted now and it’s like […] obviously like cherry picked, […] but on TikTok like other women saying, okay, they’ll only date a guy, if he’s, if he’s like this or like that’ [Young person, P5].

As this young person’s description reveals, assumptions about women’s attraction to men are based on a narrow set of physical characteristics, which are ‘like this or like that’. This sentiment directly echoes Incel ideologies regarding women’s selection of male partners, which is often taken from eugenics concepts privileging whiteness and hyper-masculine traits (Kay, 2021). As this logic claims, women prioritize men’s physical appearance, and if an individual does not fit into these narrow physical standards, they can never improve their chances of having a meaningful relationship. As another young person reported, ‘I think that women, most women in general, do not care about fucking anything other than looks when it comes to guys’ [Young person, P7]. The impacts of consuming this content can be two-pronged. First, they develop prejudices against women for the supposed rejection, and furthermore, that they will be forever undesirable and therefore struggle to develop any meaningful relationships with women, worsening isolation and poor self-esteem. This has wider mental health implications, as vulnerable young people (primarily young men) seek comradery and belonging within these groups, but which subsequently serve to isolate them further through their acceptance and belief in messaging that they will never find romantic partners.

### Theme 2: empowerment through misogynistic language

Across the four archetypes, it was apparent that the widespread proliferation and framing of toxic misogyny online had led to the normalization of misogynistic content. Examination of the content underscored how themes and tropes previously confined to Incel platforms, particularly those concerning male grievances, self-improvement activities (‘looksmaxing’), and analysis of women’s behaviors, have now become ubiquitous among male behavior in schools, as reported by the school leaders, and in the collected online content.

#### Relationships

Relationships and dating advice were presented on themes that can be clearly linked to the Incel tropes of women having unrealistic physical standards, collectively desiring the most attractive alpha males, leaving other men behind in the sexual marketplace (Menzie, 2022, p. 73). For example, one young person reported, ‘I found studies that showed that 90% of women will reject the guy who’s five foot four, despite any other things about them…I’m like what the hell? Why do I get treated differently than this guy? And it turns out the reason is because of looks.’ [Young person, P7].

Throughout the analyzed content, the four accounts primarily featured posts centered around dating and relationships that reflected these views. These posts included advice on how to attract women, strategies for seduction, and critiques of female behavior using stereotypical and pseudo-psychological analysis. There was a strong emphasis on masculinity, strength, and self-improvement, alongside the presentation of traditional gender roles and hierarchies within relationships as desirable. While some content addressed male behavior, a significant portion transitioned into specific videos that propagated negative and misogynistic tropes about ‘how women think.’ Critical posts such as ‘the truth about female nature...’ and ‘understanding the female narcissist’ portrayed relationships as transactional and perpetuated damaging stereotypes regarding expectations of sex.

This rhetoric highlights the growing phenomenon of ‘Incel 2.0’ where young men were finding empowerment through the language of Incel, using this rhetoric to articulate their contemporary frustrations in more popularized settings. The presentation of these viewpoints through cultural mediums, such as inspirational content, memes, and parodies, served to camouflage the underlying toxic and violent misogyny at its heart. As an interviewee noted:

I mean you can look at trends in meme, recent meme culture to say that it’s becoming more common. I mean you see there’s like a ‘maidenless’ meme now. People going around like saying, ‘oh like you’re, are you maidenless’ it’s like a newer meme means and yeah, (…) that’s kind of an incel thing really. Because it’s, it’s implying a hierarchy, status type of thing. And that’s implying stuff about women’ [Young person, P4].

**Figure 6:**
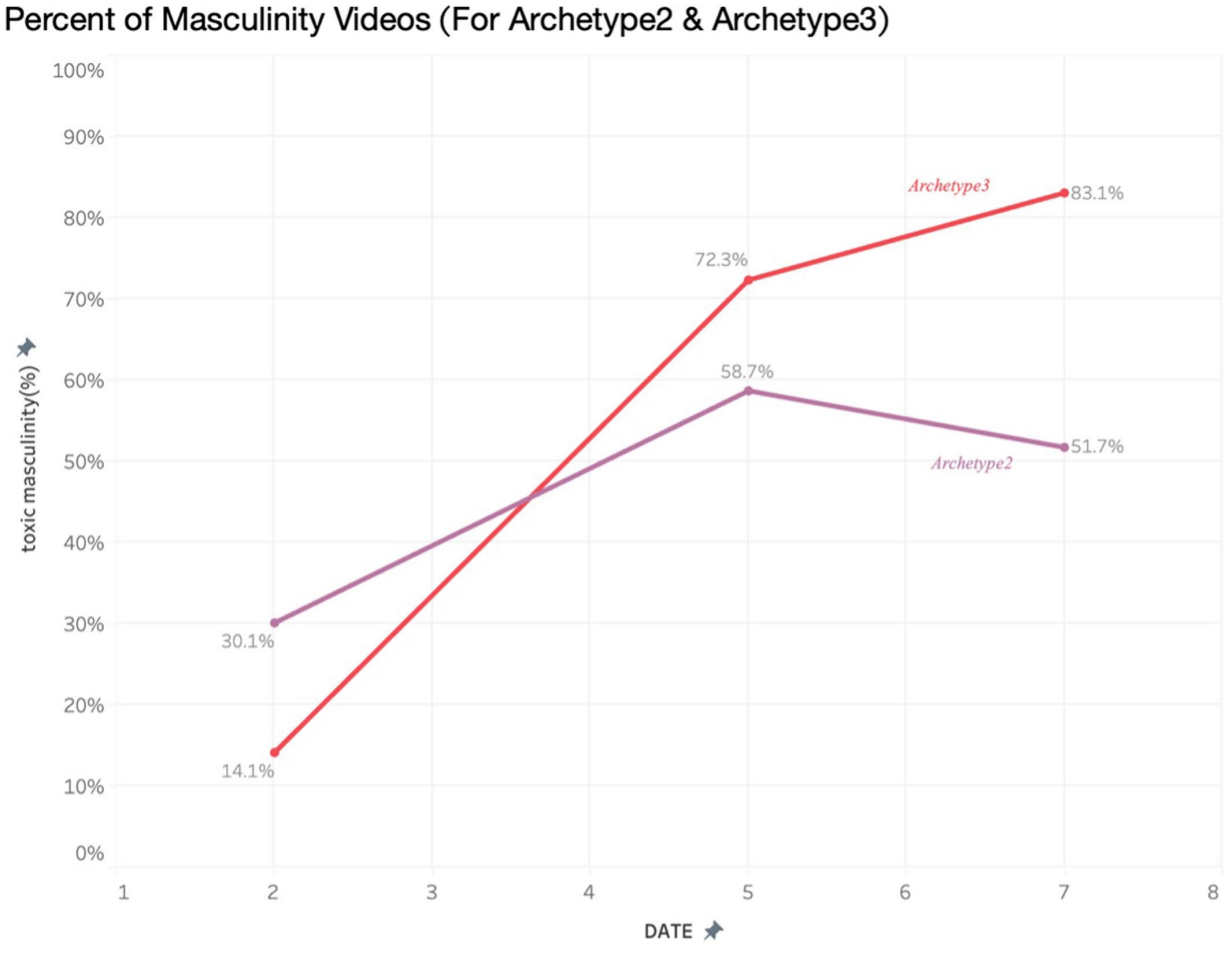
Percent of masculinity videos (For Archetype 2 & Archetype 3).

The very act of swiping up to receive ever more doses of this hateful content again masks its toxicity, having a micro-dosing effect that is at first unnoticeable (see Figure 6). As our interviews with young people further highlighted, this content permeates various online spaces to such an extent that pinpointing moments of indoctrination or influence becomes nearly impossible; “It’s like memes, you cannot really think ‘when was the first time you saw this meme’. It’s just everywhere.’. [Participant 4].

#### Strength and masculinity

Another recurring theme observed in the video clips shared across the four accounts, frequently tied to dating guidance, was the presentation of aspirational content promoting an exceedingly narrow conception of masculinity. This portrayal emphasized attributes such as physical strength, sexual prowess, and immense wealth.

The frequent cooccurrence of these two themes of relationships and masculinity formed a mutually reinforcing feedback loop, wherein videos focusing on masculinity and strength often appeared alongside those offering relationship and dating advice. These themes then intersect in the mindset of young people, as one reflected:

A lot of people you know, they’re like, F**k, you know, I’m a teenager. Like, dude, I don’t have a girlfriend, I’m trying to maximize… with like self-improvement ... you sort of go down this rabbit hole, it happens … It’s almost like political extremism… [and] you start off with like self-improvement and all that because you’re insecure, you don’t really get that many girls or you don’t get girls at all.

Here, we see typical Incel self-improvement terminology, such as ‘maximize’, which is employed to note the pressures on young men pushing them deeper into a right-leaning political ideology, thus encouraging them toward narrow and constrictive versions of masculinity. For young people, particularly those who have body-based anxieties, this can compound both their own body dysmorphia, whilst also serving to accentuate their loneliness and isolation by emphasizing how only traditional masculine stereotypes will be successful in relationships.

### Theme 3: impact of normalizing toxicity

#### Changing peer relationships

Evident across the accounts from teachers and young people, and through video evidence, was the pervasiveness of this content and how online platforms sanitize and present it to young people in easily digestible and entertaining formats. This has a micro-dosing effect, saturating youth cultures with radical misogyny and toxic content, profoundly influencing how young people interact with each other. Significantly, this online engagement is fuelling anger and discontent among and between young people, where both sides feel more polarized. As one young person reported:

‘Women don't take men's issues seriously, like they just think that they're like the biggest victims in the world. Like they don't care. Like they genuinely don't. They'll just be like, I have to worry about when I go outside. And I have to assume all men are rapists.’ [Young person, P7].

In contrast, girls reported being further victimized, as one school leader reported, ‘From our student voice work, some of the things that that the girls said to me is that social media allows men to think they can criticize us, our appearance, our personality, etc.’ [SL3].

Young people’s accounts highlight the blurring of the line between extremism and tolerance, describing how online platforms sanitize, using humor to normalize sexist language online; ‘But that was a humor form. I wasn’t like seriously using it … like a stupid joke.’ [Young person, P1] and, ‘a lot of is... a creative writing exercise (...) they usually do not mean it...’ [Young person, P4]. Participants also recognized how this humor led to normalization: ‘...we got normalized to it with these shock factor websites (...), which sends you deeper more depressed stuff because it’s like ‘what’s gonna shock me at this point?’...’ [Young person, P4].

This increased exposure also led to a lack of awareness regarding their impacts toward their female peers, as one safeguarding lead described:

It’s almost a lack of awareness, once you speak to the kids, they know it’s wrong, but they almost don’t realize it when they say it. And it’s almost because they see it so much on social media, it’s just normalized now. [SL2]

School leaders reported how they directly linked the observed rise in misogyny within schools with advancements in technology and increased access to online content. Regarding online sexual content specifically, one school leader remarked:

We did some work looking the number of students who have access to social media, and we’re looking at the high 80s every year that have got access to this material online, so the link between the two (social media and misogyny) is definitely significant. [SL4]

These issues intersect with ongoing challenges regarding digital sexual violence and IBSH. Reflecting on discussions with young people receiving unsolicited explicit images online, a school leader commented:

It’s happening out of school, so they’re not reporting it. They don’t, they are like ‘Well, who cares? Like it’s just one of those things’. So it’s sort of battling a cultural shift, a social shift in just you know what’s normal … So we’ve tried to work with them. No, no, that’s not normal. That shouldn’t happen. You shouldn’t accept that. [SL2]

There was a concern that the normalization of this behavior meant that there was a lot of underreporting of abuse and harassment, which was further changing relationships between young people. Across their student cohort, school leaders reported being aware of an ‘undercurrent of abuse’ and how the proliferation of misogynistic comments online had led to a greater confidence in some pupils to reproduce and copy these phrases. As young people further reported, ‘Eventually, it is going to be normal, like terminology that you stay quiet about things you know, but you just do not really know how to say it out loud.’ [Young person, P6].

#### Isolation and pre-existing vulnerabilities

It was notable amongst the vast majority of the young people that we spoke to that mental health concerns seemed to co-occur with their exploration of more radical, misogynistic content online, echoing prior research that identified these online communities as a way of seeking companionship and a group identity (Preston et al., 2021). As one user described, ‘I am lost (…) and directionless (…) I just want to fit in … my purpose is (…) mostly just to feel bad with other people who are feeling bad …’ [Young person, P3]. This highlights how young people may not actively seek out these ideologies but rather, are being algorithmically offered hateful or misogynistic content, as others reflected, ‘…you start off with self-improvement and all that because you are insecure…’ [Young person, P5].

Young people described their sense of isolation, the lack of awareness and the absence of support; ‘… when I was in school there wasn’t really enough done to address with these men, lonely men.’ [Young person, P2]. Invalidation was also identified as a contributory factor to their situation, compounding their sense of hopelessness or anger. This suggests that a process is in place where young men, seeking community and understanding, or as an interviewee explains, a ‘need for friendship and like for socializing’ are finding an answer, endorsement and empathy in these communities. The complexities of these contextual and environmental factors were recognized by teachers:

It's not necessarily all linked to misogyny. I think it’s more mental health issues. They're starting younger and we certainly saw a rise in kind of the Andrew Tate thing last year in boys … there was a pocket of boys that were talking about it all the time. [SL5]

As other studies have identified (Costello et al., 2022; Sparks et al., 2024), the most isolated and vulnerable individuals are those most at risk of indoctrination. This was echoed by one safeguarding lead:

‘We've got a very small number of boys who are absolutely fixed on their views and we struggle to kind of get them to see anything else… And I think with regards to that, the more high risk students there, I think really that's where we do need some more specialist support within that. And I think that's something that I think for us at the minute in the city we're struggling with’.

This corresponds to concerns from teachers that neurodivergent adolescent men are a high-risk group and the reported prevalence of autism and neurodivergence in misogynist online communities (Tirkkonen and Vespermann, 2023). As participants themselves recognised, ‘most people (…) on these servers (…) have a form of autism. I am autistic (…) I did not tested for autism, I just 100 per cent know I’m autistic.’ [Young Person, P1]. However, it is important to emphasize that this is a controversial topic with mixed results, calling for care and sensitivity to the complexities and the need to be ‘extremely cautious when making generalizations and associating violence with autism’ (Williams et al., 2021, pp. 395–396). There is a range of contested factors contributing to this association, which need to be considered in any endeavor to address and support. In the case of autism (as the most frequently cited example by community participants in our research and other studies), the experience of neuro-difference impacts on social relationships, cognitive processes (thinking and learning), emotions, and sense of self, often affecting mental and physical health.

The changes associated with adolescence and the transition to adulthood are particularly difficult for autistic young people, many of whom experience anxiety, depression, and loneliness. Gender plays a critical role in identity formation, and for neurodivergent cisgender boys seeking identification and confirmation of their masculinity, peer pressures to conform to social expectations of gendered behavior are felt particularly strongly. Gender becomes a performative effort to fit in so much so that masking and assimilation are reported as having negative impacts on mental health for autistic adolescents (boys and girls), albeit with different behaviors associated with gender differences. While autistic girls are prone to internalizing their differences (associated with underdiagnosis) (Mandy et al., 2012; Lai et al., 2015), boys are more likely to exhibit challenging or distressed behavior, masking their mental health difficulties. All of this contributes to a toxic environment in which hate ideology can thrive (particularly misogyny) through difficulties with peers (e.g., bullying and female relationships). Recent research (Tirkkonen and Vesperman, 2023) has identified the processes whereby online forums can have a more detrimental impact on neurodivergent young people. The negative themes identified in the Tirkkonen and Vesperman’s study correspond to many of those identified in our interviews, particularly in terms of hopelessness, body image and futures.

#### Changing school policies

In the review of the video content and school leaders’ responses, it became evident that existing approaches for dealing with the behavioral impacts of the consumption of online misogyny have limitations. The staff at one boys’ school wondered if their ‘Incel problem’ was a subsequent attempt on the boys’ part to swing the pendulum back after movements like ‘everyone’s invited’, which tackled sexual violence in schools. Instances like these highlight the problematic pedagogical and educational approaches available for working with boys that often ‘re-essentialize masculinities and embed limited assumptions about boys’ (Equimundo, 2020). Indeed, as one school leader reflected, previous pedagogical approaches had a negative effect on boys’ willingness to engage:

I think whenever we talk about Andrew Tate, I think the defenses come up and the boys think they're gonna get beaten with a stick again and we're trying to tell them how they should think, and how they should behave. But I think the more we flood them with the positive role models and raise aspirations around it, I think is a key strategy to use.

As young people also reflected, responses from teachers served to further entrench their views due to negative responses; ‘But the teacher was always so pissed off at me. You could see like how angry she was at me. And she would not look at me (…) she would not even acknowledge me…’ [Young Person, P3]. Less salient are more nuanced discussions regarding the ways in which Incel discourse might offer a means for young men to voice a fear of loss of control at a time that is very bleak for all young people. As one young person commented ‘…men are oppressed (…) isolated (…) I find some sort of solstice in guys like Andrew Tate…’ [Young Person, P2] Pedagogically embedded interventions, which address the ways in which we might include boys in the common goal of actualizing healthy practices and relationships on and offline are more limited still. As one young man pointed out, ‘I’m told everything I cannot do and cannot be’ [Young Person, P1] without being given positive role models or alternatives. It is in these gaps in understanding and educational approaches that we observed how extremist, misogynistic content has become saturated in popular youth ecosystems. Responses up until now that involve specific moderation of individual videos and influencers are, therefore, limited in their ability to tackle the problem. There are a plethora of other influencers—or in(cel)encers—and content promoting misogynistic messaging to greater and lesser degrees that now populate the feeds of teenage boys. This content is now simply a symptom of a much larger cultural phenomenon: the popularizing of technologically facilitated misogyny in the form of Incel 2.0 through mainstream social media platforms.

## Discussion

Our results highlight how social media algorithmic recommender systems are increasingly putting young people at risk. Recommender systems are exposing young people to harmful material, which, through the affordances of these platforms, is presented as entertainment in young people’s feeds. As a result, hateful ideologies and misogynistic tropes are becoming normalized in young people’s behaviors both online and offline. We found that the algorithms privilege more extreme material, and through increased usage, users are gradually exposed to more misogynistic ideologies, which are presented as ‘soft’ or ‘humorous’ cultural forms. Similar to other studies, which have looked topics such as the alt right (Ribeiro et al., 2021), or self-harm (Amnesty International, 2024), our research found that after only 5 days of TikTok usage, there was a 4-fold increase in the level of misogynistic content being presented on the ‘For You page; on TikTok. In this way, toxic, hateful or misogynistic material is pushed to young people and exploiting adolescents’ existing vulnerabilities. Boys who are seeking community, are suffering from poor mental health, bullying, or who are neurodivergent are at heightened risk. The fact that these harmful ideologies, such as sexism and misogyny, are now normalized amongst young people means that behaviors are seeping into their everyday interactions. The proliferation of misogynistic ideas and language has moved off screens and into schools, where they are frequently enacted in mainstream youth culture. Young people increasingly exist within digital echo-chambers, which normalize this rhetoric and subsequently impact their individual and social development.

This study was carried out in a UK context, and as such, our recommendations are specifically focused on the UK context. We first presented our preliminary findings of this research at the Association of School and College Leaders Annual Safeguarding Conference for pastoral leads and school leaders from across England. The wealth of responses highlighted the growing concerns regarding the impacts of digital engagement on adolescent development. In addition, the number of media reports on this issue and high-profile parent groups (including Smartphone Free Childhood) who have called for better understanding and increased restrictions on smartphones highlights the level to which these concerns are now embedded within cultural conversations. As others have highlighted, responses include for social media companies to actually enforce their age restrictions, which they and others (Farah and Milmo, 2023) argue is currently woefully unenforced. Looking beyond restriction, we consider below how to tackle this growing phenomenon, including young people in these conversations and respecting their own digital freedoms and rights:

### Holding industry accountable

Our case study of online misogyny reveals how hateful content is algorithmically offered to young people and how these online processes impact the school environment. In light of these impacts, the Tech industry needs to be held responsible for these harmful algorithmic processes. This means not just focusing on removing individual harmful content or videos, but on the underlying structures and processes that they have developed that perpetuate these types of echo-chambers. Pressure needs to be applied so that big tech companies, like TikTok, address algorithmic harm and prioritize the wellbeing of young people over profit.

### Amplifying youth voice

Including boys in the conversation through peer-to-peer mentoring to tackle gender-based violence in schools and young people’s online behaviors. This ensures that young people are included in these discussions and co-create new codes of conduct. An example, which has been developed alongside this research, is the Mentors in Violence Protection (MVP) program trialed by Education Scotland. Here, older pupils are trained to lead sessions and mentor younger pupils around topics like online misogyny. Older pupils are empowered into leadership roles, they are embedded in the school ecosystem and can continually monitor young students’ progress. This has a further impact of supporting a wider cultural change within schools and among young people. As many teachers reflected, the most powerful advocates are young people themselves, and that good quality student voice work on the issue is central to making an impact. As one teacher reported, ‘they always find it faintly embarrassing when a person of my age tries to keep up with kids on social media.’

### Supports for staff to engage with both pupils and parents with critical digital literacy

Schools are now met with extreme challenges around the impacts of social media consumption, but many times, teachers and parents are not equipped with the knowledge, understanding, or previous life experience to support young people. In particular, so much happens online, out of school hours, that they cannot control. Even though it is now common for no smartphones to be allowed during school hours, many still see the ramifications of all that online behavior within their setting. In particular, there also needs to be further education for teachers and parents around how the algorithms work and increased dialogue between parents and staff, with a recognition that parents also have a role to play.

## Conclusion

These findings highlight how the affordances of social media platforms, particularly recommender systems of TikTok, actively amplify and direct harmful content. We found that the algorithms privilege more extreme material, and through increased usage, users are gradually exposed to more and more misogynistic ideologies, which are presented as entertainment ‘soft’ cultural forms. Social media’s sophisticated technological affordances provide a potent indoctrination effect, and this micro-dosing on highly toxic content is leading to the saturation of extremist misogynistic ideas among young people, with highly detrimental effects on their wellbeing.

We echo the recommendations made in a report to ASCL in January 2024 regarding our findings from working with their school leaders, of a need to consider variegated approaches to digital prohibition vs. digital literacy. Recent evidence highlighting the ability of young people to circumvent age restrictions online (Farah and Milmo, 2023), and the rapid emergence of new or copycat platforms, emphasizes the limitations of legislation to respond in a digital age. As we have seen in the uses of social media across this study, there is a plethora of issues which are driven by the affordances of the platforms themselves. These relate to changes in personal relationships; interpersonal and romantic, u-loops and the lack of spontaneous cultural inputs, which creates a more polarized society. We argue that the way in which this material is accessed and offered to young people—by way of algorithmic processes that alter and distort how content is consumed—leads to deeply unhealthy developmental shifts in the way young people think and interact with others.

Advocates have repeatedly called for educational initiatives, with Facebook whistleblower Frances Haugen stating that young people are often left to navigate social media-driven issues on their own without adult support. Nevertheless, education-based interventions and support for teachers’ issues have been slow and woefully unable to keep pace with changing technologies and the implications they bring for young people. Blame is then often incorrectly placed on teachers to address issues, which primarily take place outside of school hours, or on the young people themselves. To effectively embed critical digital literacy in schools, we recommend a linked-up approach between safeguarding, school leadership, teachers, and parents—and if at all possible, the tech industry at large—in order to support young people with key skills to recognize radicalization and be critical about toxic online material as they transition to adulthood.

### Limitations and future recommendations

This research was not without limitations. The authors acknowledge that the relatively small sample size of interview participants is limiting in terms of representation and generalizability. Additionally, although multiple (five) researchers were involved in the coding and spot checking of the video data, the collection *via* interviews was done by one researcher (with experience of working with the community and building relationships of trust). These potential limitations could be addressed in previous research.

## Data Availability

The datasets presented in this article are not readily available because of the sensitivity of the qualitative personal data collected during this study. Requests to access the datasets should be directed to c.shaughnessy@ucl.ac.uk.

## Footnotes

^1^The researcher joined these forums using one of their anonymous Discord accounts.

^2^Note that further details of the recruitment process for the interviews (including specific forums and groups) have been withheld to protect the privacy of interviewees, as well as to prevent the further popularization of these forum. More detail about the specific recruitment processes can be obtained from the authors upon request.

^3^The identification of misogynistic content adhered to established criteria outlined in prior research (Shushkevich and Cardiff, 2019), encompassing various elements, such as stereotype perpetuation, objectification, dominance assertion, derailing, sexual harassment, threats of violence, and discrediting. Videos meeting any of these criteria were classified as containing misogynistic content.
